# Surveillance of febrile patients in a district and evaluation of their spatiotemporal associations: a pilot study

**DOI:** 10.1186/1471-2458-10-84

**Published:** 2010-02-20

**Authors:** Kin-wing Choi, Ngai-sze Wong, Lap-yip Lee, Shui-shan Lee

**Affiliations:** 1 Department of Medicine, Alice Ho Miu Ling Nethersole Hospital, Tai Po, Hong Kong; 2 Stanley Ho Centre for Emerging Infectious Diseases, School of Public Health and Primary Care, the Chinese University of Hong Kong, Hong Kong; 3 Accident and Emergency Department, Alice Ho Miu Ling Nethersole Hospital, Tai Po, Hong Kong

## Abstract

**Background:**

Fever is an undifferentiated clinical feature that may enhance the sensitivity of syndromic surveillance systems. By studying the spatiotemporal associations of febrile patients, it may allow early detection of case clustering that indicates imminent threat of infectious disease outbreaks in the community.

**Methods:**

We captured consecutive emergency department visits that led to hospitalization in a district hospital in Hong Kong during the period of 12 Sep 2005 to 14 Oct 2005. We recorded demographic data, provisional diagnoses, temperature on presentation and residential location for each patient-episode, and geocoded the residential addresses. We applied Geographical Information System technology to study the geographical distribution these cases, and their associations within a 50-m buffer zone spatially. A case cluster was defined by three or more spatially associated febrile patients within each three consecutive days.

**Results:**

One thousand and sixty six patient-episodes were eligible for analysis; 42% of them had fever (>37°C; oral temperature) on presentation. Two hundred and four patient-episodes (19.1%) came from residential care homes for elderly (RCHE). We detected a total of 40 case clusters during the study period. Clustered cases were of older age; 57 (33.3%) were residents of RCHE. We found a median of 3 patients (range: 3 - 8) and time span of 3 days (range: 2 - 8 days) in each cluster. Twenty five clusters had 2 or more patients living in the same building block; 18 of them were from RCHE.

**Conclusions:**

It is technically feasible to perform surveillance on febrile patients and studying their spatiotemporal associations. The information is potentially useful for early detection of impending infectious disease threats.

## Background

With the lessons learnt from the severe acute respiratory syndrome (SARS) epidemic in 2003 and heightened awareness on emerging infectious diseases, e.g. avian influenza [1,2], there has been a clear demand globally on effective infectious disease surveillance mechanisms that can achieve early event detection and health situational awareness. As a result, automated syndromic surveillance systems have emerged in recent years, and its applicability has been evaluated in various studies. Most of these reported studies focused on emergency department visits, and data were grouped into different syndromes (e.g. respiratory) [3-5]. Through continuous data collection, secular trend on incidence of specific syndromes and hence thresholds to define abnormal signals were established [6,7]. However, there was no consistent approach on syndrome grouping and methodology of data analysis.

Fever is an undifferentiated clinical feature that is often present in patients with infections. Incorporation of fever as a syndrome may enhance the sensitivity of surveillance systems. Furthermore, by mapping of residential areas of febrile patients and studying their spatiotemporal associations, it is possible to detect case clustering that herald imminent threat of infectious disease outbreaks in the community.

## Methods

We attempted to study the spatiotemporal associations of emergency department visits that presented with fever and hospitalized, and identified clustering of cases to predict occurrence of outbreaks.

Tai Po (147.84 km^2^) is a district in the Hong Kong Special Administrative Region of China and it has a population of about 300,000 residents. Most of the residents in this district are living in apartment buildings with multiple (3 to over 30) storeys, a typical floor of which contains 4 to 8 flats that measure 40 to 90 m^2 ^in size.

Alice Ho Miu Ling Nethersole Hospital (AHNH) is the only public hospital that provides acute medical services to the residents of Tai Po. Following the outbreak of SARS in 2003, all emergency department visits are screened for the body temperature, and patients with fever are segregated in a separate waiting area. We prospectively captured consecutive emergency department visits of AHNH that led to hospital admissions during the period of 12 Sep 2005 to 14 Oct 2005, both days inclusive. For each patient-episode, we obtained data on demographics, temperature on presentation, provisional diagnoses, destined ward for admission and residential location. These data were available from the Clinical Management System, an electronic platform that is in place at all public hospitals in Hong Kong for retrieval of archived patient information (e.g. emergency department attendance record, hospital discharge summary, results of investigations etc.) and clinical management (e.g. request of laboratory tests, prescription of medications, etc.). The provisional diagnoses were recorded in a text field and we went through them manually to decide whether they represented infections or not. In AHNH, each clinical department owned its designated wards and patients were assigned their destined wards by their presenting illnesses. In addition, designated isolation wards for contagious infections (e.g. open pulmonary tuberculosis) were available under the medical and paediatric departments. The information on destined wards would facilitate outbreak investigation and infection control when clustering of febrile patients was found. As we focused on the detection of case clustering within Tai Po, we excluded patient-episodes with residential addresses without this district.

Each textural residential address was transformed to x and y coordinates in Hong Kong Grid 1980. Using Geographical Information system (GIS) technology, data were created and geocoded in the form of point data. Each patient-episode was represented by a point and the rest of data were recorded in an attribute table. The creation of point data enabled spatial visualization and analysis of these patient-episodes.

In studying the spatial associations among patient-episodes, we assumed that the most usual activity space of each patient was around 50 meters (m) from the respective residential location, and a 50 m buffer (i.e. a 50 m radius circle) was created for each point. If there was overlapping of two or more activity spaces, it might indicate associations among these patients in terms of their illnesses. Thus, spatially adjoining buffers were then merged to form a bigger buffer. Each three consecutive days was used as a temporal unit when analyzing the spatial associations. The data were left-censored by the emergency department attendance date.

Numerical variables were summarized as either mean and standard deviation (SD) or median and range, whichever was appropriate. Categorical variables were summarized as percentages. Unpaired Student's t-test and Chi-square test were used for univariate analyses. We defined fever as an oral temperature above 37°C or equivalent, and a case cluster as 3 or more spatially associated patient-episodes with fever during each 3-day period. The spatial growth of each cluster was monitored in GIS and the corresponding time span was recorded. The data points of the final map have been randomly distributed within a radius of 50 meters, which did not therefore show the actual residential locations of patients in the study. ESRI ArcGIS version 9.2 was used for the GIS related data processing and analyses, and SPSS version 13 was used for other statistical analyses.

This study was approved by the Survey and Behavioural Research Ethics Committee, the Chinese University of Hong Kong.

## Results

We identified 1538 patient-episodes (by 1413 patients), and successfully geocoded the residential addresses in 1320 of them. After exclusion of patient-episodes with residential locations without the Tai Po district, 1066 patient-episodes were eligible for analysis. Of these, 204 (19.1%) were from residential care homes for elderly (RCHE).

**Figure 1:**
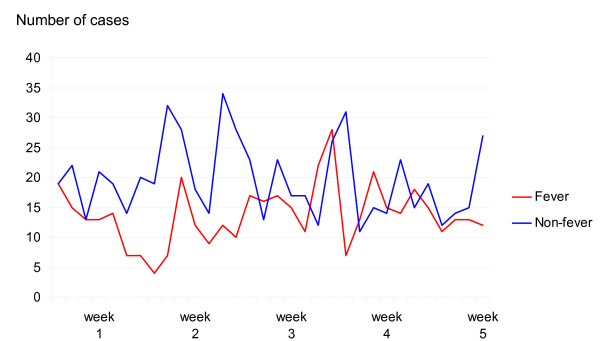
**Daily admission rate through the emergency department and distribution of patients with fever**.

Among the 1066 patient-episodes, 448 (42.0%) were febrile on presentation (Figure 1). Younger patients, especially those aged less than 12 years, were more likely to present with fever (Table 1). Two hundred and nine patients with fever were provisionally diagnosed to have infections at the emergency department (47.2% vs. 7.8% of patients with normal temperature). Table 2 summarizes the top 5 diagnoses in these 2 groups of patients.

**Table 1:** Univariate analysis on (1) patients with fever vs. patients with normal temperature; (2) patients within clusters vs. non-cluster patients.

(1) Patients with fever vs. patients with normal temperature (n = 1066)			
	**Fever** **(n = 448)**	**Normal temperature** **(n = 618)**	**Odds ratio (95% confidence interval)**

Age (mean ± SD)^‡^	51.7 ± 31.7	60.8 ± 25.7	-

Age ≤12 years (number, %)^‡^	87 (19.4%)	54 (8.7%)	2.52 (1.75, 3.62)

Age 13 years to 64 years (number, %)	166 (37.1%)	235 (38.0%)	0.96 (0.75, 1.23)

Age ≥65 years (number, %)^‡^	195 (43.5%)	329 (53.2%)	0.68 (0.53, 0.87)

RCHE* residents (number, %)	98 (21.9%)	106 (17.2%)	1.35 (1.00, 1.84)

Infections diagnosed (number, %)^‡^	209 (47.2%)	48 (7.8%)	10.55 (7.44, 14.95)

**(2) Patients within clusters**^**† **^**vs. non-cluster patients (all had fever, n = 448)**			

	**Cluster** **(n = 171)**	**Non-cluster** **(n = 277)**	**Odds ratio (95% confidence interval)**

Age (mean ± SD)^‡^	56.2 ± 30.7	49.0 ± 32.0	-

Age ≤12 years (number, %)	27 (15.8%)	60 (21.7%)	0.68 (0.41, 1.12)

Age 13 years to 64 years (number, %)	63 (36.8%)	103 (37.2%)	0.99 (0.66, 1.46)

Age ≥65 years (number, %)	81 (47.4%)	114 (41.2%)	1.29 (0.88, 1.89)

RCHE* residents (number, %)^‡^	57 (33.3%)	41 (14.8%)	2.88 (1.82, 4.57)

Infections diagnosed (number, %)	85 (49.7%)	124 (44.8%)	1.20 (0.82, 1.76)

*RCHE: residential care homes for elderly.

^†^Defined as 3 or more spatially associated patient-episodes with fever during each 3 consecutive days.

^‡^p-value < 0.05

**Table 2:** Top 5 diagnoses made at the emergency department

Patients with fever	Patients with normal temperature
Fever, not otherwise specified	Chest pain, not otherwise specified
Decreased general condition	Decreased general condition
Pneumonia/chest infection	Dizziness, not otherwise specified
Sepsis, not otherwise specified	Chronic obstructive airway disease
Chronic obstructive airway disease	Dyspnea, not otherwise specified

**Figure 2:**
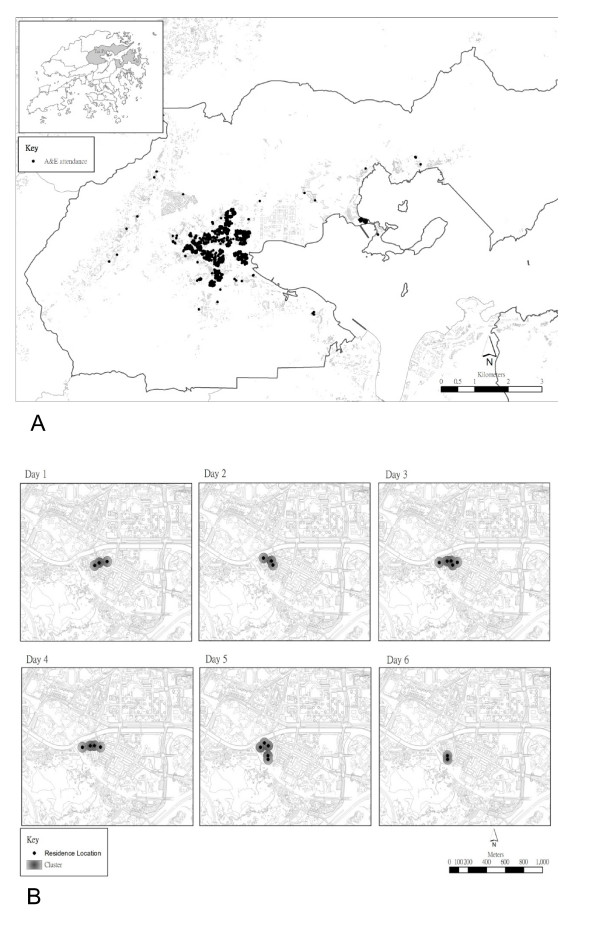
**Spatial display of the cases**. 2A: Distribution of individual cases. 2B: An example on spatiotemporal evolution of clusters.

We identified 40 clusters (comprising 171 patient-episodes) during the study period. Most clusters contained 3 patients (23 clusters had 3 patients, 5 clusters had 4, 4 clusters had 5, 5 clusters had 6, 1 cluster had 7 and 2 clusters had 8; median cluster size: 3 patients). Twenty five clusters (62.5%) had two or more patients living in the same building block; of these, 18 (45.0%) had two or more patients living in the same RCHE. Clustered cases were of older age, and residents of RCHE were responsible for 33.3% of the cases (Table 1). Spatiotemporal evolution of clusters was monitored and visualized graphically by the GIS (Figure 2), and they lasted for a median of 3 days (range: 2 - 8 days). Subsequent analysis of the results on microbiological investigations revealed evidence of parainfluenza virus as a probable etiological cause in one cluster and respiratory syncytial virus in another; both clusters were associated with RCHE.

## Discussion

Previous studies on syndromic surveillance of emergency department visits usually focused on the temporal trend of specific clinical syndromes (e.g. respiratory, gastrointestinal, etc.) [3-7]. While GIS is often incorporated into syndromic surveillance systems nowadays, it is mainly used to visualize burden of specific clinical syndromes or diseases in different parts of a city or country [8,9]. Spatiotemporal analysis by means of the space-time scan statistics has been gaining popularity in recent years [10,11]. Our surveillance methodology provides additional information on the spatiotemporal associations and hence possible clustering of cases.

We used fever as an indicator of infections in this study. Though non-specific, it has the advantage of being measurable objectively. Also, clinical manifestations of emerging infectious diseases can be protean, and there is a potential chance of underestimating the degree of disease burden in community if we concentrate on a limited number of clinical syndromes in conducting syndromic surveillance. On the other hand, fever is an undifferentiated clinical feature that occurs commonly in most infectious diseases. We expect improvement on sensitivity of outbreak detection when incorporating fever as a syndrome into surveillance systems. We selectively recruited emergency department visits that led to hospitalizations for three operational reasons. Firstly, these patients represent a more severe end of spectrum of illnesses; if we detect a cause of impending infectious disease outbreak that can lead to severe illnesses, the impact on public health will be greater. Secondly, when a cluster is detected, it is possible for us to carry out the necessary investigations with respect to the etiology of the hospitalized patients and verify whether there is a genuine outbreak. Thirdly, early identification of case clusters that are potentially associated with outbreaks will facilitate infection control management within the hospital, e.g. cohorting of cases [12]. The definition of case cluster in this study is somehow arbitrary. We tried to use a common sense approach in deriving this definition: a cluster of three or more persons with the same clinical syndrome is often regarded as epidemiologically significant in outbreak investigation; a time frame of 3 days (to define temporal association) is within the incubation period of most infectious diseases [13]; and an activity space of 50 m in radius should meet the living conditions for most of the people living in the district under our study. We examined the effect of variation of the buffer zone radius and duration of the temporal unit on the number of clusters detected. The results were summarized in Table 3. Taking this and the aforementioned rationales into account, we considered that a buffer zone radius of 50 m and temporal unit of 3 consecutive days be appropriate for our data analysis.

**Table 3:** Effect of variation on radius and temporal unit on the number of clusters detected.

	Duration of temporal unit		
**Buffer radius**	***1 day***	***3 days***	***5 days***

***25 m***	2	21	35

***50 m***	6	40	51

***100 m***	31	25	17

Application of GIS allows direct visualization on the geographic pattern and spatiotemporal evolution of case clustering. This can give us an immediate perception on the extent of the problem, and it may also help to assess the efficacy of public health interventions on the control of an established outbreak.

There are several important findings in this study. Firstly, younger patients were more likely to have fever as presenting illness to emergency departments. Elderly people were not more likely to have fever; but if they did, they tended to cluster. Secondly, among the 40 clusters identified in this study, 25 had 2 or more patients living in the same residential premises. Spreading of infections by a novel pathogen in residential premises could result in potentially devastating consequences; a major outbreak of SARS in a housing estate in Hong Kong is a vivid reminder on the possible sequelae [14,15]. Thirdly, a significant proportion of patients (19.1%) were residents of RCHE, and they contributed to 45% of clusters identified in this study. Residents of RCHE are often susceptible to infections because of their advanced age and presence of co-morbidities. Outbreaks of infectious diseases in RCHE are common, and result in a significant burden to the healthcare system. The high percentage of elderly patients and residents of RCHEs in this cohort reflects their health seeking behavior and the population aging in Hong Kong. In this locality, healthcare is mainly provided by the Hospital Authority, a government funded organization which provides territory-wide, hospital-based medical services to the citizens in Hong Kong. Moreover, a substantial proportion of residents in RCHEs are physically dependent and hospitalized repeatedly because of the underlying medical conditions. Therefore, there is often a tendency for the care providers to send these patients to emergency departments for clinical management. Together with the lower threshold for hospital admission, results in this study were skewed towards the elderly population. In Hong Kong, fever surveillance at sentinel RCHE (percentage of residents that are febrile) has been in place since 2007. Otherwise, detection of clustering relies solely on passive reporting by staff of RCHE. Aging is a challenge to health authorities in most industrialized countries and development of a surveillance system for detection of severe infections in geriatric patients would be an important step to safeguard their health [16].

Traditionally, temporal association of cases with compatible clinical syndromes formed the basis on definition of outbreaks [6,17]. Spatiotemporal analysis adds another dimension on the evaluation of syndromic data for early event detection and health situational awareness. Advances in space-time scan statistics and availability of a computer software resulted in major breakthroughs in this area [10,11]. Its fundamental principle is the application of a scanning window that moves across space and/or time. For each location and size of the window, the number of observed and expected cases is counted. The window with most excess of observed cases (i.e. a cluster) is noted. The statistical significance of this cluster is thus evaluated. This method provides an objective estimate on the likelihood of clustering, and it has been applied in the study of geographical distribution of diseases over a period of time [18,19], investigations of outbreaks and their progression [20,21], and analysis of syndromic data from emergency department visits [10,22], among the others. As an example, in the spatiotemporal analysis of syndromic data ''fever/flu'' from the New York City Emergency Department Syndromic Surveillance System [10], 4 signals suggestive of possible clustering were detected over a period of one week when using the geographical coordinates of the patients' residential zip codes, a radius of 5 km and the time span of 7 days. This and other studies on the spatiotemporal analysis of syndromic data from emergency department visits often focused on more specific syndrome groups (e.g. diarrhea, respiratory illness, etc.) [22,23]; this is in contrast with our approach of fever surveillance, which relies on a non-specific indicator of infections. The essence of space-time scan statistics is detection of the excess number of cases under study in a pre-defined time frame and area; our method provides complementary information on spatiotemporal associations among individual cases. Limited by differences in the populations under study, healthcare infrastructure, syndromic data in question and methods of analysis, direct comparison of results in this study and literature reports is difficult.

Taking into account the relatively short study period, the number of clusters detected by our surveillance methodology was substantial. This result was expected, as our surveillance method was based on a sensitive and non-specific indicator of infection. Nevertheless, since all the recruited cases were hospitalized, their clinical course and results of relevant investigations were readily available for review. Thus, it was not difficult to verify whether these clusters represented genuine outbreaks. We consider it be technically feasible to build an automated surveillance system through expansion of the functions on our existing electronic platform. As of the time of writing, development of a territory-wide electronic communicable disease information and surveillance system is under way in Hong Kong.

This study has several limitations. Firstly, since we used the residential addresses of our patients for spatiotemporal analysis, we were unable to detect potential outbreaks that occurred in other important community indwelling facilities like schools or child care centers. Secondly, the system was not automated at the time of study. Thirdly, as a pilot study, we have not investigated the case clusters in a real time manner. Fourthly, we have not categorized our patients based on their provisional diagnoses at emergency department to refine the definition of case cluster. However, as illustrated in Table 2, the provisional diagnoses were often non-specific. This phenomenon is in part explained by the skewing of our results towards elderly population, and it is not uncommon for them to present with multiple, vague symptoms. Finally, due to constraints in resources, we were only able to analyze the data from a one-month period. Hence, the effect of seasonal variation on incidence of various infections was not addressed. For example, there are typically 2 influenza peak seasons in Hong Kong, namely July to August and December to February of next year.

## Conclusions

In conclusion, it is technically feasible to perform surveillance based on simple and objective data on patients' temperature and studying the spatiotemporal associations among febrile patients. Further study is needed to test the applicability of our method on other syndromic data, refine the definition of case clustering, define the signal to noise ratio, determine effect of inclusion of other data (e.g. admission diagnosis) on the alert threshold, test the effect of system automation, and evaluate its impact on the prevailing healthcare systems.

## Competing interests

The authors declare that they have no competing interests.

## Authors' contributions

KWC participated in the study design and data collection, and drafted the manuscript. NSW participated in the study design and performed the spatiotemporal analysis of the data. LYL participated in the data collection. SSL conceived of the study, participated in its design, performed statistical analysis, and helped to draft the manuscript. All authors read and approved the final manuscript.

## Pre-publication history

The pre-publication history for this paper can be accessed here:

http://www.biomedcentral.com/1471-2458/10/84/prepub
